# The effect of lowering salt intake on ambulatory blood pressure to reduce cardiovascular risk in chronic kidney disease (LowSALT CKD study): protocol of a randomized trial

**DOI:** 10.1186/1471-2369-13-137

**Published:** 2012-10-19

**Authors:** Emma J McMahon, Judith D Bauer, Carmel M Hawley, Nicole M Isbel, Michael Stowasser, David W Johnson, Rachael E Hale, Katrina L Campbell

**Affiliations:** 1 Princess Alexandra Hospital, 199 Ipswich Road, Woolloongabba, Brisbane, QLD, 4102, Australia; 2 University of Queensland, Brisbane, 4072, Australia

**Keywords:** Dietary sodium, Chronic kidney disease, Blood pressure, Cardiovascular disease, Arterial stiffness, Clinical trial, Patient compliance, Taste disturbance

## Abstract

**Background:**

Despite evidence implicating dietary sodium in the pathogenesis of cardiovascular disease (CVD) in chronic kidney disease (CKD), quality intervention trials in CKD patients are lacking. This study aims to investigate the effect of reducing sodium intake on blood pressure, risk factors for progression of CKD and other cardiovascular risk factors in CKD.

**Methods/design:**

The LowSALT CKD study is a six week randomized-crossover trial assessing the effect of a moderate (180 mmol/day) compared with a low (60 mmol/day) sodium intake on cardiovascular risk factors and risk factors for kidney function decline in mild-moderate CKD (stage III-IV). The primary outcome of interest is 24-hour ambulatory blood pressure, with secondary outcomes including arterial stiffness (pulse wave velocity), proteinuria and fluid status. The randomized crossover trial (Phase 1) is supported by an ancillary trial (Phase 2) of longitudinal-observational design to assess the longer term effectiveness of sodium restriction. Phase 2 will continue measurement of outcomes as per Phase 1, with the addition of patient-centered outcomes, such as dietary adherence to sodium restriction (degree of adherence and barriers/enablers), quality of life and taste assessment.

**Discussion:**

The LowSALT CKD study is an investigator-initiated study specifically designed to assess the proof-of-concept and efficacy of sodium restriction in patients with established CKD. Phase 2 will assess the longer term effectiveness of sodium restriction in the same participants, enhancing the translation of Phase 1 results into practice. This trial will provide much-needed insight into sodium restriction as a treatment option to reduce risk of CVD and CKD progression in CKD patients.

**Trial registration:**

Universal Trial Number: U1111-1125-2149. Australian New Zealand Clinical Trials Registry Number: ACTRN12611001097932

## Background

**Figure 1:**
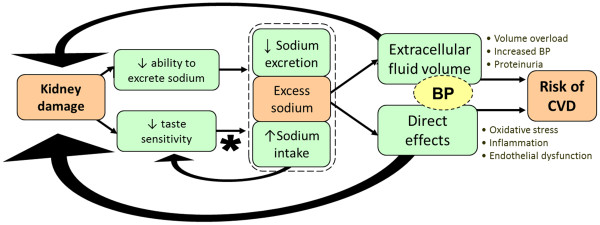
**Simplified diagram of the relationship between excess sodium, kidney damage and risk of CVD.** Excess sodium in CKD is caused by decreased sodium excretion & high sodium intake (*influenced by food supply and preference – potentially mediated by taste sensitivity). This increases cardiovascular risk not only via altered extracellular volume & blood pressure (BP) but also through direct toxic effects on blood vessels.

Cardiovascular disease (CVD) is the leading cause of mortality in the chronic kidney disease (CKD) population, with CKD patients being 5–10 times more likely to die of CVD than to progress to end-stage kidney disease (ESKD) [1,2]. Dietary sodium intake has been associated with numerous modifiable risk factors for CVD in CKD including increased blood pressure (BP), volume overload, left ventricular hypertrophy, inflammation and endothelial damage [3-6] (Figure 1). Indicators of kidney damage, proteinuria and albuminuria have also been associated with sodium intake [6,7], and are key risk factors for subsequent all-cause and cardiovascular mortality [8,9]. However, despite evidence implicating dietary sodium in the pathogenesis of CVD in CKD, quality intervention trials in CKD patients are lacking.

Several meta-analyses of dietary sodium intake and blood pressure (BP) have been published, consistently demonstrating that a reduction in dietary sodium intake leads to reduced BP, particularly in hypertensive populations [10-14]. Unfortunately most studies included in these analyses exclude patients with kidney disease. Since sodium balance is primarily the role of the kidney, it is highly likely that CKD patients have a reduced ability to excrete the high sodium intake that is typical of a Western diet, increasing susceptibility to the adverse effects of excessive dietary sodium [15,16]. This may partially explain why hypertension is so prevalent in CKD, with data indicating that up to 70% of CKD patients are hypertensive [17].

An issue with translation of research in sodium restriction into practice is poor dietary adherence. This has been reported in several research trials [18-21]. Furthermore, data indicate approximately 80-90% of CKD patients consume more than 100 mmol of sodium per day [19-21], meaning that most do not adhere to the target set by evidence-based practice guidelines [22-26]. Achieving sodium restriction can be difficult in research and in practice. Therefore, it is vitally important to establish the feasibility of achieving adherence to sodium restriction in the clinical setting, as well as exploring the efficacy of this intervention.

Dietary sodium shows great promise as a modifiable risk factor for reduction of cardiovascular risk and risk of renal function decline in CKD, but high quality evidence is lacking. There is a need for a research trial showing the efficacy of sodium restriction for reducing the risks of CVD and CKD progression in CKD. Further, the effectiveness of recommendations to restrict sodium should be assessed to ensure the results are translatable to practice.

## Methods/design

### Study aim

The aim of the LowSALT CKD study is to examine the impact of reducing dietary sodium intake in decreasing cardiovascular risk in CKD patients. Our primary hypothesis is that a low sodium intake (LSI) (60 mmol/day) in patients with stage III or IV CKD will be independently associated with decreased ambulatory BP compared to a moderate sodium intake (MSI) (180 mmol/day). Our secondary hypothesis is that LSI (60 mmol/day) will result in the following outcomes compared with MSI (180 mmol/day): decreased arterial stiffness; decreased proteinuria and albuminuria; decreased extracellular fluid and markers of volume overload and thirst.

### Study design and setting

**Figure 2:**
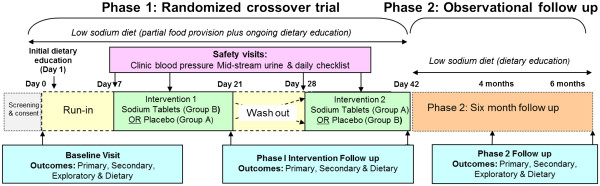
LowSALT CKD study flowchart.

The LowSALT CKD study is a single-centre double-blind randomized cross-over trial (Phase 1) with a prospective observational follow-up (Phase 2). Phase 1 entails a 7-day run-in period followed by two, 2-week interventions (Figure 2). Participants follow a low sodium diet (goal: 60 mmol/day) together with either moderate sodium intake (achieved via slow-release sodium tablets providing 120 mmol sodium/day) or placebo (low sodium intake) for 2 weeks duration, and crossover to the other intervention with a 1-week washout in between (Figure 2). These levels of sodium intake represent two extremes of typical sodium intake in the Western diet, with the moderate sodium target (180 mmol sodium/day) representing that found in observational studies [19,27,28] and the low sodium target (60 mmol/day) resembling a level that meets even the strictest evidence-based practice guidelines specific to CKD [29]. Phase 2 involves a further 20-week observational period, where participants will be encouraged to adhere to a low sodium diet, and outcome measures repeated at 4 and 6 months (after commencement of Phase 1).

### Ethical considerations

Ethical approval has been obtained through Metro South Human Research Ethics Committee and University of Queensland Human Research Ethics Committee.

### Target population and eligibility criteria

The target population for the trial will be stage III and IV non-dialyzed CKD patients. Eligibility criteria will include hypertensive, CKD stage III or IV (GFR 15–59 ml/min/1.73 m^2^) non-dialyzed, non-transplanted patients under the care of a nephrologist at the Princess Alexandra Hospital; aged ≥ 18 years with systolic BP 130–169 mmHg and diastolic BP ≥ 70 mmHg; non salt-wasting CKD (as diagnosed by nephrologist); prescribed ≤ 20 mmol sodium per day via medications; not pregnant or breastfeeding; life expectancy > 6 months; no current involvement in other intervention study and mental capacity to adhere to the study protocol (as indicated by medical team).

### Recruitment of participants

Potential participants will be identified through the outpatient clinic lists at the Princess Alexandra Hospital (PAH). To confirm that a potential participant’s blood pressure is within range, BP will be measured once in seated position when the participant attends clinic and previous BPs will be collected from the medical notes. Once a potential trial participant has been screened as meeting the eligibility criteria, recruitment by informed consent will be sought either at the time of the clinic visit or at a later date (providing additional time to consider the information). Once consented, patients will undergo 24-hour ambulatory blood pressure (ABP) measurement at baseline to confirm eligibility. Patients with 24-hour BP ≤ 130/70 will be discontinued from the study due to the potential risk of hypotension with sodium reduction. Patients and the treating doctors will be asked to keep the anti-hypertensive medications unchanged for the duration of the trial within bounds of patient safety.

### Dietary counseling

All patients will be counseled to follow a low sodium diet for the entire trial duration. Initial dietary education will occur face-to-face during run-in, with ongoing education during follow up visits and phone calls (Table 1). Topics covered will include: target sodium intake, general guidelines for following a low sodium diet, reading labels, foods and drinks high in sodium and lower sodium alternatives (individualized to participant’s usual dietary habits), low salt cooking methods and recipe ideas. In addition, participants will be provided with written dietary education materials (based on established resources used in CKD and heart failure patients to reduce sodium intake [30,31]). During Phase 1, participants will also be provided with a selection of low sodium foods to facilitate adherence to a low sodium diet (Table 1).

**Table 1:** Strategies to facilitate sodium restriction

**Phase 1**	**- Dietary education (individualized) by accredited practicing dietitian**
	- Focus on replacing high sodium foods with low sodium alternatives (keeping energy intake stable).
	- Initial education in run-in with written materials provided
	- Ongoing education during follow up visits and weekly phone calls.
	**- Partial food provision**
	- Low sodium frozen pre-prepared meals (maximum 1 per day)
	- Snack foods, breakfast cereal, cheese and bread
**Phase 2**	**- Ongoing dietary education**
	- Monthly via phone calls
	- No food provided in this phase

### Randomization

An external statistical consultant will undertake the computer-generated randomization for the order of the intervention in blocks of 8, blinded to the investigators. To ensure all investigators and participants are blinded to the treatment, study medications are packaged off-site by an external pharmacy with a generic label as medication 1 and 2 for the first and second intervention.

### Intervention

As per Figure 2, following a 7 day run-in period, participants will enter ‘treatment’ (moderate sodium arm, 180 mmol/day) or ‘control’ (low sodium arm, 60 mmol/day) for 2 weeks duration and cross-over to the alternate treatment. During ‘treatment’ participants will receive 120 mmol sodium /day via slow-release sodium chloride tablets (4 × 10 mmol tablets t.d.s; Slow Sodium, Novartis Pharmaceuticals Australia Pty Ltd), in addition to 60 mmol dietary intake. During ‘control’, participants will receive white placebo capsules (4 × capsules t.d.s, size 1D cellulose capsules, Pharmaceutical Packaging Professionals Pty Ltd). A washout period (1 week duration) will precede intervention two; this period was chosen as previous research indicated this is the duration needed to achieve sodium balance [32]. During washout, participants will continue to consume a low sodium diet (goal 60 mmol/day).

### Primary outcome - ambulatory blood pressure

24-hour ABP was selected as the primary outcome as it is the most accurate reflection of BP, with measurements over an entire 24-hour period indicating diurnal variation, while avoiding error due to white-coat or masked hypertension [33-35]. Data will be measured at time points as per Figure 2 and will be analyzed as mean 24-hour BP, daytime BP and night BP.

ABP will be measured using the TM-2430 Ambulatory Blood Pressure Monitor (A&D Medical, Australia). This device is a non-invasive lightweight portable device that is validated in accordance with British Hypertension Society protocol, with A/A rating [36]. ABP measurements will be conducted according to the ‘Practice Guidelines of the European Society of Hypertension’ for clinic, ambulatory and self BP measurement [33] with systolic and diastolic BP recorded every 20 minutes during the day (6 AM to 10 PM) and 30 minutes at night (10 PM to 6 AM) [33,37,38]. The left arm will be used for measurement, except when medical history indicates an existing claudication. The measurement arm will be kept consistent during the study. ABP monitoring will be considered adequate if at least two-thirds of the measurements taken over the 24-hour period are satisfactory [33]. Participants will be trained on the use of the 24-hour BP monitor when they attend the clinic to have measurements taken, and will be asked to record their sleep and wake times during the BP monitoring period. Investigators and patients will be blinded to ABP results.

### Secondary outcome

#### Arterial stiffness

Arterial stiffness will be assessed by carotid-femoral Pulse Wave Velocity (PWV), which is the current gold standard for non-invasive measurement of aortic arterial stiffness [39], and by radial Pulse Wave Analysis (PWA), which derives the augmentation of the central pulse waveform via measurement at the peripheral (radial) artery. Both measurements will be taken with SphygmoCor^TM^ CPV (AtCor Medical) which employs applanation tonometry to measure the shape and velocity of the pulse wave, with good repeatability and reproducibility [40].

To ensure accuracy and reproducibility, measurements will be taken in the morning after fasting blood samples. Participants will be provided with a standardized breakfast (breakfast cereal and/or muesli bars) and will be asked to abstain from tobacco and caffeine at least 4 hours before the measurement. PWV will be assessed by measuring carotid and femoral artery wave-forms sequentially over the common carotid and femoral arteries. Radial PWA will be used to estimate central pulse pressure and augmentation index. Both PWV and PWA measurements will be measured in duplicate. Validity of the measurements will be assessed as per the SphygmoCor manual [41], with an operator index of >80 indicating a good quality measurement for PWA and a standard error <10% of the PWV value indicating a good quality measurement for PWV. Where significant disagreements between the measurements exist, the measurement will be repeated until measurements within 10% of each other are obtained.

#### Urinary protein and albumin

24-hour urine samples (gold standard for measuring proteinuria and albuminuria) [42] will be used in this trial. Participants will be provided with bottles for collection and educated on correct collection procedure in accordance with standard protocol. Storage and processing of the urine samples will also be carried out according to standard protocol. Both participants and investigators will be blinded to the urinary results.

### Fluid status

Fluid status, a predictor of cardiac alterations such as left ventricular hypertrophy [3], will be indicated by bio-impedance spectroscopy (BIS). This will be assessed by the Body Composition Monitor (BCM) (Fresenius Medical Care, Germany), which determines total body and extracellular water by measuring electrical resistance across 50 frequencies using electrodes placed on the wrist & foot, (5 to 1000 kHz) [43,44]. BIS is a quick, non-invasive method to assess fluid status, validated against gold standard dilution methods [45]. The BCM has been shown to be an accurate indicator of fluid change when compared to body weight before and after dialysis sessions in haemodialysis patients [44]. Measurements will be performed in accordance with the manufacturer’s instructions [46]. Patients with stents or pacemakers will be excluded from this assessment. Participants and investigators will be blinded to the BIS results.

### Secondary outcome - xerostomia index (thirst)

Thirst will be measured by the Xerostomia Index (XI) [47-49]. The XI is a score summated a subjective 11 item questionnaire [47] measuring the severity of the symptoms of dry mouth across one of five responses (‘never’, scoring 1; ‘hardly ever’, 2; ‘occasionally’, 3; ‘fairly often’, 4; and ‘very often’, 5)’. Each individual’s responses will be scored and summated to give a single XI score [47].

### Secondary outcomes: serum metabolic markers

Serum blood samples will be stored for analysis of markers of inflammation (CRP), adiponectin (total and high molecular weight), markers of renin-angiotensin-aldosterone system stimulation (renin, aldosterone, angiotensin-converting-enzyme) and a marker of fluid overload (NT-proBNP) in addition to the standard tests taken in practice (e.g. full blood count and urea and electrolytes). Blood samples will be collected after overnight fast and drawn from a peripheral vein while participants are seated. Blood will be centrifuged within 30 minutes after blood collection. After separation, plasma will be transferred using a graduated plastic disposable pipette into color coded storage vials and placed in freezer storage boxes in a −80°C freezer. Additional serum will be stored for the analysis of novel risk factors in the future. Participants and investigators will be blinded to the serum outcomes.

### Adherence/dietary intake measures

**Figure 3:**
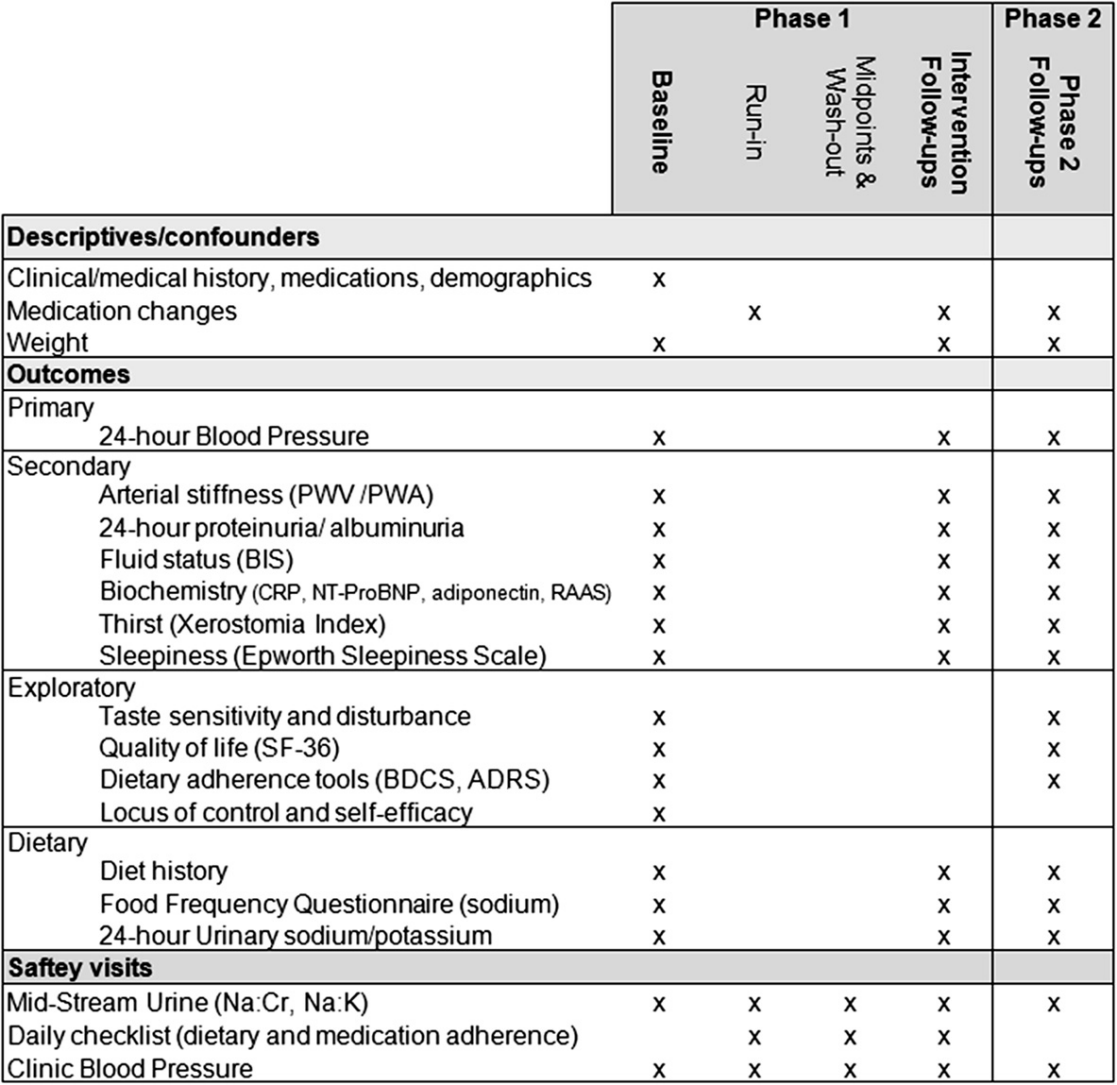
LowSALT CKD study data collection schedule.

A multi-method approach to measuring dietary sodium intake was chosen to employ both objective and self-reported methods, optimizing accuracy of dietary intake estimation, while minimizing participant burden [50]. The current gold standard for dietary sodium intake measurement is repeated 24-hour urinary sodium excretion [51,52]. Due to the increased participant burden involved in 24-hour urine collection, 24 hr collections (at baseline and all follow up points) will be supplemented with mid-stream urine collections occurring weekly in Phase 1 and at follow up points in Phase 2 (see Figure 2). Mid-stream urinary sodium has recently been demonstrated to have good agreement with 24-hour urinary sodium in CKD [53,54], and the collection of 24-hour samples concurrently with mid-stream samples will allow for further exploration of the agreement between these measures. Mid-stream urine samples will be analyzed for sodium, creatinine and albumin concentrations (Figure 3).

In addition to objective measures, a diet history and food frequency questionnaire (FFQ) will be used to measure dietary intake. In the diet history method, information about what is consumed over an extended period of time is collected using open-ended questions [55]. This is considered an ideal method for capturing ‘usual intake’ over a specified period of time [56]. To minimize the time burden and participant recall bias, which typically limit the use of the diet history, this trial will use a self-administered diet history (Wollongong method [56]) with forms mailed to participants in advance and verified by a dietitian interview. The FFQ, which is designed specifically to quantify sodium intake [52], will also be mailed with the diet history. Participants will complete these forms at home and bring them to their appointment where information will be verified by the study dietitian using food models and visual prompts to quantify intakes. Dietary histories will be entered into FoodWorks 7 (Xyris Software, Version 7.0.2915) using the Nuttab 2010 database (for general unbranded foods and known recipes), AusBrands 2012 database (where specific brand is known) and AusFoods 2012 (for convenience or pre-prepared foods where recipe is unknown). The FFQ will be scored as per Charlton protocol [52].

The final method for measuring intake will be a daily checklist, used throughout the entire 6 weeks of Phase 1. This is designed to capture intake of foods likely to contribute to a high sodium intake (from unpublished data of CKD patients at the PAH, (Longitudinal Assessment of Multiple Discrete Atherosclerotic Risk factors in Kidney Disease (Landmark) Study, Isbel N et al.), as well as daily record of study medication. The information on this checklist will be verified for completeness and accuracy. Adherence to the study medication will also be measured by pill count at trial end. Medication changes will be recorded, with all efforts made to avoid change of anti-hypertensive medications during the six week trial.

### Phase 2

Phase 2 of the trial (observational arm to 6 months after baseline visit) aims to assess long-term changes in outcomes and adherence to a sodium-restricted diet, as well as establishing common barriers and enablers to achieving dietary sodium restriction in this group. In this phase, barriers and enablers to adherence will be measured via four self-administered questionnaires. Self-efficacy will be measured using the health-related general self-efficacy scale [57] and health-related locus of control using the Multidimensional Health Locus of Control [58]. Beliefs about dietary compliance scale (BDCS) [59] and attitudes to dietary recommendations scale (ADRS) (modified from [60]) will be assessed. Results from the locus of control and self-efficacy scales will be used to identify predictors of dietary adherence. Results from the beliefs about dietary compliance (BDSC) will be used to identify specific barriers and enablers to dietary adherence. Results from the attitudes to dietary recommendations (ADRS) will assist in identifying specific recommendations with which participants find it difficult to adhere.

Another outcome specific to Phase 2 is taste assessment of the five basic taste senses: salty, sweet, bitter, sour and umami. Evidence suggests that hedonic liking and taste sensitivity of salty taste can be modified with adherence to a low sodium diet [61], however this is yet to be established in the CKD population. A further gap in the literature is whether restriction of dietary sodium could affect the other four taste senses; (sweet, bitter, sour and umami). In accordance with the International Standards Organization method of investigating sensitivity of taste [62], participants will be presented with 2 ml solutions of substances which represent each of the five basic taste qualities (and which are found ubiquitously throughout the food supply) and asked to identify each taste as well as the strength of the taste on a 10-cm visual analogue scale. This enables assessment of ability to detect each taste and the degree of sensitivity, measured at baseline and phase 2 follow up visits (Figure 2).

CKD patients have been found to have significantly decreased quality of life (QoL) with QoL decreasing as GFR declines [63]. Hypertensive patients have also been found to have reduced QoL scores when compared with normotensives [63]. Restricted sodium intake may increase quality of life by reducing symptoms associated with fluid overload, such as sleep apnea and edema. Therefore, quality of life (QoL) will be assessed by the Short Form-36.

Overall, Phase 2 aims to translate the promising indications from basic science into clinical practice demonstrating the effect of dietary sodium on blood pressure, risk factors for CKD progression and cardiovascular disease.

### Statistical analysis

The primary outcome will be change in 24-hour SBP from matched pairs of study subjects (by cross-over design) from MSI to LSI. Prior data indicate that the mean response of matched pairs is a 5 mm Hg change in BP with a standard deviation of 7 mm Hg (unpublished data from Todd et al. [64]). If the true difference in the mean response of matched pairs is 5 mmHg, 23 subjects across both treatments (to give 46 data sets) will be required to give a power of 90% with error of 0.05. Accounting for an adjustment factor of 1.56 (drop out of 20%; drop-in of 0%), a total of 37 patients will be recruited and pass run-in.

Analyses will be undertaken by analysis of covariance (ANCOVA) adjusting for baseline values using STATA (Version 12 [65]). To test for time order effects, both the treatment and treatment order will be included in the model. If the treatment order is not found to be significant, treatment group will be left in the model. Probability values of <0.05 will be considered significant. Test for normality of the distribution of each variable will be performed separately for each treatment group.

## Discussion

This trial will provide evidence on the effects of reducing dietary sodium on cardiovascular risk factors and risk factors for kidney function decline in participants with mild to moderate CKD. We anticipate this intervention, a low sodium intake (60 mmol/day) (compared to usual sodium intake (180 mmol/day), will result in significant reductions in 24-hour BP, arterial stiffness, excess fluid and inflammation, all of which have been shown to be predictors of cardiovascular abnormalities and/or morbidity and mortality in CKD patients [3,66,67]. The outcomes of this research will provide valuable proof-of-concept data for future research in dietary management of CVD risk in CKD, with the hypothesis being that therapeutic control of sodium intake will reduce CV disease progression and events in CKD patients.

A number of potential confounding factors will be considered in this trial. Potassium intake reduces BP; therefore changes in potassium intake between the high and low sodium periods may confound the trial results [68]. As such, participants will be counseled to keep potassium intake stable. In addition potassium intake will be measured using multiple methods to strengthen validity of the data (24-hour urinary excretion, mid-stream sodium-to-potassium ratio, diet history). Weight also directly affects BP [69], so all efforts will be made to keep weight stable during the 6 week trial and weight will be measured at all follow up visits as per Figure 2. GFR may directly affect serum biochemical markers, therefore estimated GFR (eGFR) will be assessed concurrently with other biochemical markers.

There are several attributes to the study design that we believe will ensure robustness of the results. By employing the use of sodium chloride tablets versus placebo while blinding both the participants and the investigators to the treatment we will be avoiding a confounding effect of changes in other dietary nutrients, while also reducing risk of bias. The use of gold standard measurements such as 24-hour BP further strengthens the study. Conducting this trial in stage III-IV CKD allows us to investigate a strategy that may potentially delay or prevent adverse outcomes (such as CVD or ESKD) early in the disease. Furthermore, we believe the provision of dietary counseling by an accredited practicing dietitian with an individualized approach will assist dietary adherence. In addition, the multi-method approach to measuring dietary intake will ensure that dietary adherence will be measured as accurately as possible. Finally, the long-term follow up will enable us to assess long-term effects of sodium restriction, whilst also observing barriers and enablers to dietary adherence that may be targeted to aid sodium restriction.

## Conclusion

The outcomes of this research will provide valuable proof-of-concept data for a future study of dietary management of CVD in CKD, with the hypothesis that therapeutic control of sodium intake will reduce CV disease progression and events in CKD patients.

## Competing interests

The authors declare that they have no competing interests.

## Authors’ contributions

EM participated in the trial design, developed the statistical plan and drafted the manuscript. KC conceived of the trial, and participated in its design, developed the statistical plan and helped to draft the manuscript. JB participated in the trial design, coordination and statistical plan and helped to draft the manuscript. CH, NI, MS, DJ participated in trial design. RH participated in trial design and coordination. All authors read and approved the final manuscript.

## Pre-publication history

The pre-publication history for this paper can be accessed here:

http://www.biomedcentral.com/1471-2369/13/137/prepub
